# Antifreeze protein from *Ammopiptanthus nanus* functions in temperature-stress through domain A

**DOI:** 10.1038/s41598-021-88021-0

**Published:** 2021-04-19

**Authors:** HaoQiang Yu, HongYing Zheng, Yuan Liu, QingQing Yang, WanChen Li, YuanYuan Zhang, FengLing Fu

**Affiliations:** 1 Key Laboratory of Biology and Genetic Improvement of Maize in Southwest Region, Ministry of Agriculture; Maize Research Institute, Sichuan Agricultural University, Chengdu, 611130 China; 2 College of Life Science & Biotechnology, Mianyang Teachers’ College, Mianyang, 621000 China

**Keywords:** Plant sciences, Plant biotechnology

## Abstract

Temperature stress restricts plant growth and development. Antifreeze protein (AFP) can improve plants antifreeze ability. In our previous study, the *AnAFP* gene cloned from *Ammopiptanthus nanus* was confirmed to be an excellent candidate enhancing plant cold resistance. But, AnAFP protein shared similar structures with KnS type dehydrins including K, N and S domains except ice crystal binding domain A. Here, we generated *AnAFPΔA*, *AnAFP*ΔK, *AnAFPΔN* and *AnAFP*ΔS, and transformed them into ordinary and cold sensitive strains of *E. coli*, and *Arabidopsis* KS type dehydrin mutant to evaluate their function. Expression of *AnAFPΔA* decreases cold and heat tolerance in *E. coli*, meanwhile, AnAFP enhances heat tolerance in *Arabidopsis*, suggesting that domain A is a thermal stable functional domain. AnAFP, AnAFPΔA and AnAFPΔS localize in whole cell, but AnAFPΔK and AnAFPΔN only localizes in nucleus and cytoplasm, respectively, exhibiting that K and N domains control localization of AnAFP. Likewise, K domain blocks interaction between AnAFP and AnICE1. The result of RT-qPCR showed that expression of *AnAFP*, *AnICE1* and *AnCBF* genes was significantly induced by high-temperature, indicating that the *AnAFP* is likely regulated by ICE1-CBF-COR signal pathway. Taken together, the study provides insights into understanding the mechanism of AnAFP in response to temperature stress and gene resource to improve heat or cold tolerance of plants in transgenic engineering.

## Introduction

Temperature stress including low- and high-temperature stress restricts plant growth and development. Low temperature inhibits enzyme activity and destroys membrane permeability resulting in physiological disorder, metabolic obstruction and even cell death^1,2^. Similarly, high temperature leads to wilting, accumulation of reactive oxygen species (ROS), and destruction of membrane system^3–5^. After perceiving temperature stress, plants approach genes expression change, physiological and biochemical response via signal transduction^5–8^. The ICE1-CBF-COR pathway is the most studied signaling pathway under temperature stress in plants. Under low temperature conditions, a MYC-like basic helix loop helix (bHLH) transcription factor ICE1 (Inducer of CBF expression 1) induces expression of the CBF gene. The CBF transcription factor binds to the CRT/DRE (C-repeats/dehydration responsive) element (CCGAC) and activates the expression of cold-responsive genes (COR)^9,10^. In response to high temperature stress, the energy releasing from cell physiological disorder such as membrane fluidity increasing, DNA unwinding and protein subunit dissociation triggers transcriptional changes to restore homeostasis, promoting cell survival, and elaborating longer-term responses for adaptation, growth, and development^11^.

Antifreeze proteins (AFPs) were firstly found in polar fishes, as well as insects living in freeze-zone. AFPs prevent water from freezing by adsorbing to the ice surface and stopping the growth of minute ice crystals to large crystals in a non-colligative manner, and help the organisms survive in subzero temperature environments. The distribution of AFPs in different species appears to be the outcome of a combination of independent evolutionary events, which is probably the convergent evolution or horizontal gene transfer^12^. These AFPs genes were introduced into crops to improve their tolerance to low temperature stress. Expression of antifreeze protein of winter flounder or insect (*Microdera puntipennis dzungarica*) confers the cold tolerance to transgenic spring wheat at subzero temperatures, tobacco or tomato, respectively^13–15^. Meanwhile, the transgenic plants did not demonstrate significant tolerance improvement when compared to wild-types^13–15^. The non-colligative manner of the heterologous animal AFPs, as well as their expression rate, localization, and stability, might not be suitable for cellular environments in transgenic plants^16^. However, the AFPs from overwintering plants including *Lolium perenne*, *Loliurn perenne* and *Ammopiptanthus nanus* showed higher inhibitory effect on ice growth and recrystallization than that of AFPs of fishes and insects^17–20^.

*Ammopiptanthus nanus* (*A. nanus*) is a tertiary relict plant and evergreen broad-leaved shrub distrusted in deserts in Central Asia, exhibits excellent tolerance to abiotic stress including drought, low and high temperature. In our previous study, the *AnAFP* gene was cloned from xerophyte *A. nanus*, and evaluated its cold tolerance function by ectopic expression in *Escherichia coli* (*E. coli*), tobacco and maize^21,22^. Bioinformatics analysis showed that AnAFP shared high similarity with some members of KnS type dehydrins^22^. In addition to the ice crystal binding domain (A domain) of AFPs, AnAFP also has three conserved domains of dehydrins including K, S and N (Nuclear localization sequence) domains^22,23^. In order to evaluate the function of these domains, four mutants of *AnAFP* deleting A (*AnAFPΔA*), K (*AnAFPΔK*), S (*AnAFPΔS*) and N (*AnAFPΔN*) domain were generated by overlapping PCR, respectively. In this study, these four mutants and *AnAFP* were introduced into cold-sensitive and ordinary strains of *E. coli*, as well as *Arabidopsis* mutant of KnS type dehydrin gene *AtHIRD11* to identify thermal stability of each domain under low- and high-temperature stress, respectively. Together with their subcellular localization, interacting proteins and induced endogenous expression, the regulation mechanism of the AnAFP protein in response to temperature stress was elucidated.

## Results

### Sequence of mutant genes

Through overlap PCR, the sequences of *AnAFPΔA*, *AnAFPΔK*, *AnAFPΔN* and *AnAFPΔS* were amplified from *AnAFP* (Figure S1a). Their encoding putative proteins were deleted of crystal binding domain A, and K, NLS and S domains of dehydrin, respectively (Figure S1b).

### Expression of *AnAFPΔA* increases cold sensitivity of BX04

**Figure 1:**
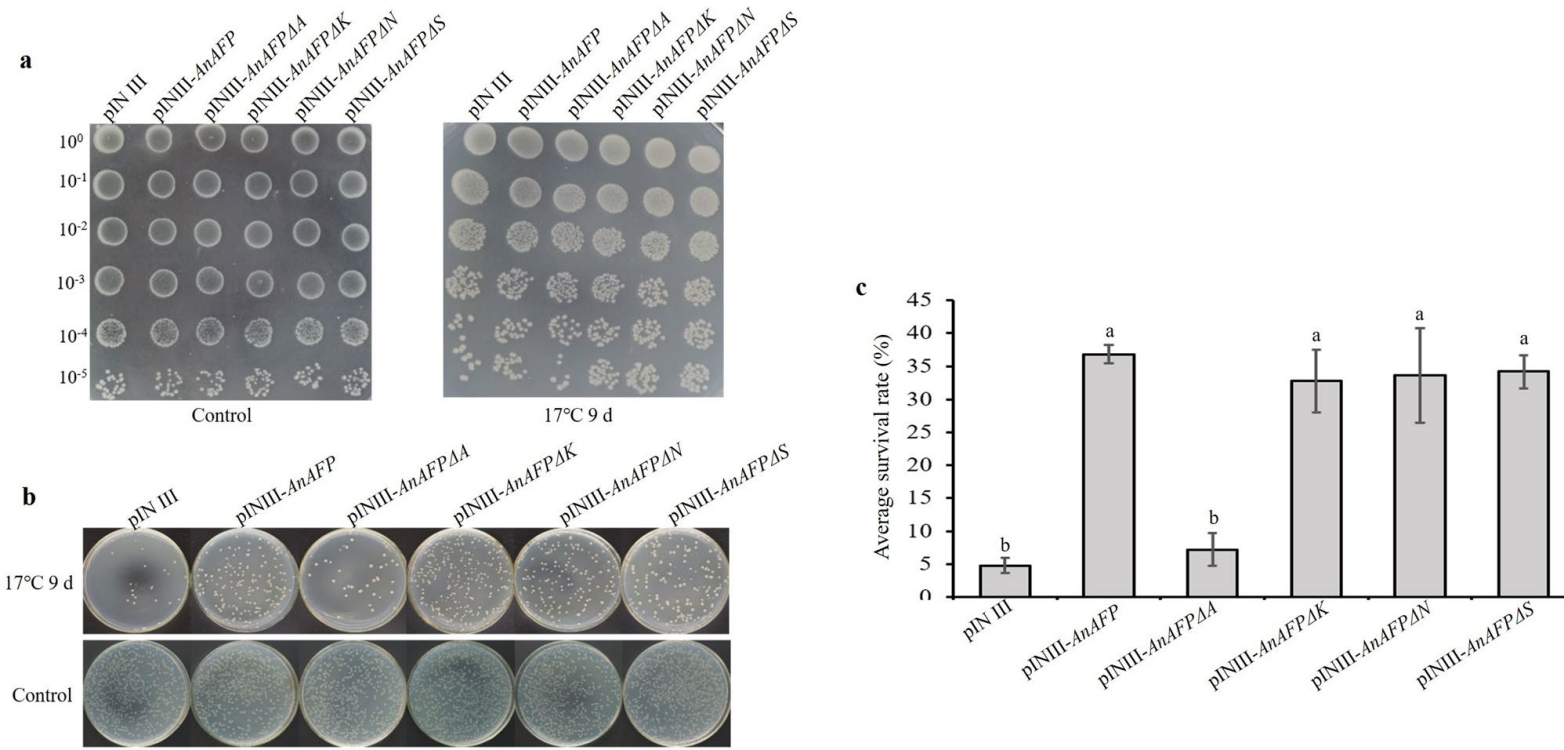
Phenotype of cold sensitive BX40 strain of *E. coli* with *AnAFP*, *AnAFPΔA*, *AnAFPΔK*, *AnAFPΔN* and *AnAFPΔS* gene under low temperature stress (17 °C) for 9 days. (**a**) Colony growth of different dilution times. (**b**) Monoclone of transformed strains. (**c**) Average survival rate of transformed strains. Lower case letter indicates the significant difference at p < 0.05 level in student’s *t*-test. The experiment was performed with three replicates. The data were presented as the mean values ± SD.

After low temperature treatment at 17 °C for 9 days, the cold-sensitive BX04 containing pINIII-*AnAFPΔA* showed growth defect and failed to form colonies with an average survival rate < 10%, which was similar to BX04 cells contain pINIII. However, the BX04 containing pINIII-*AnAFPΔK*, pINIII-*AnAFPΔS* and pINIII-*AnAFPΔN* grew vigorously and formed more colonies with an average survival rate > 30%, respectively, which was similar to BX04 cells with pINIII-*AnAFP* (Fig. 1). These results suggest that domain A is a functional domain of AnAFP protein related to cold tolerance.

### Expression of *AnAFPΔA* increases heat sensitivity of BL21

**Figure 2:**
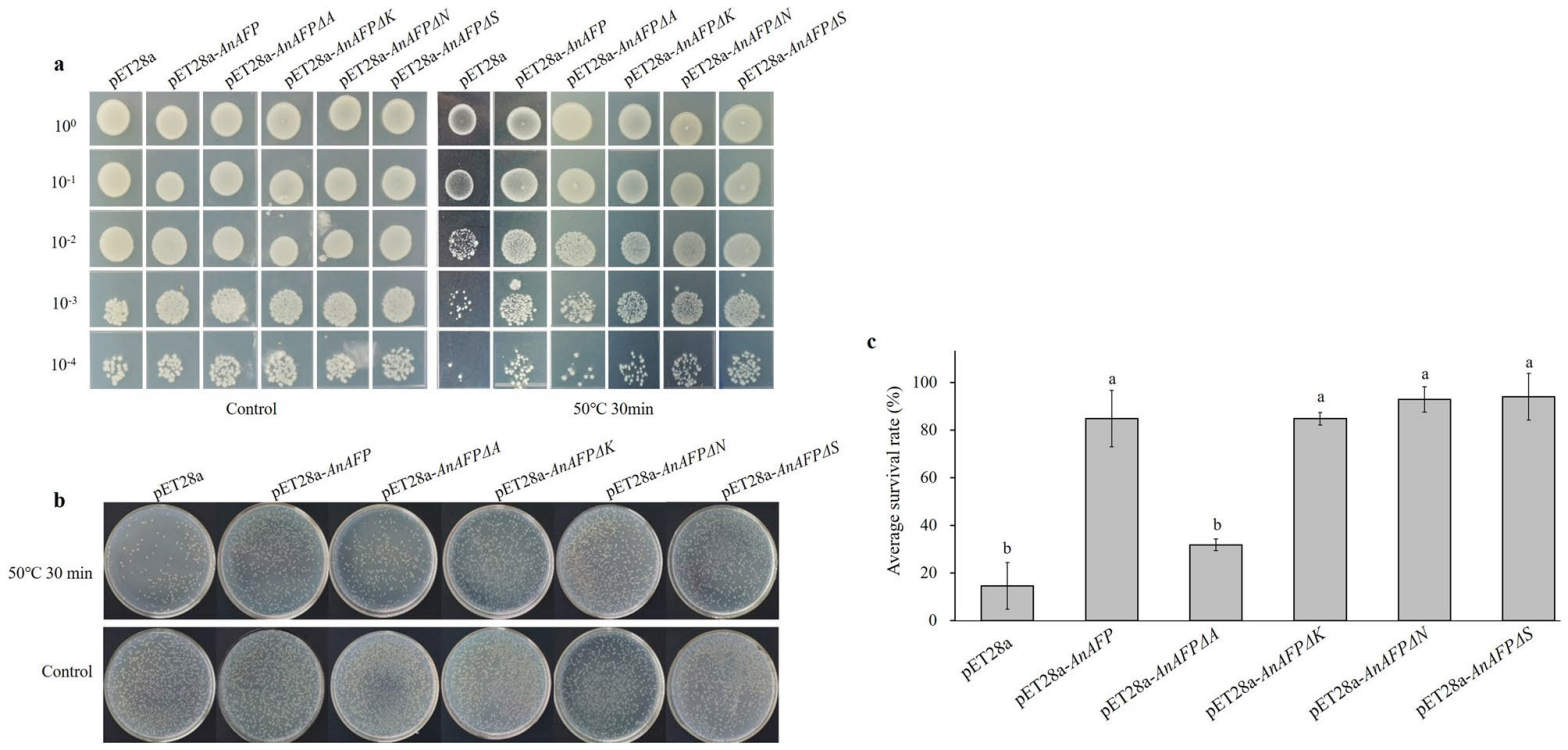
Thermotolerant phenotype of *E. coli* BL21strains transformed by *AnAFP*, *AnAFPΔA*, *AnAFPΔK*, *AnAFPΔN* and *AnAFPΔS* gene under heat stress (50 °C) for 30 min. (**a**) Colony growth of different dilution times. (**b**) Monoclone of transformed strains. (**c**) Average survival rate. Lower case letter indicates the significant difference at p < 0.05 level in student’s *t*-test. The experiment was performed with three replicates. The data were presented as the mean values ± SD.

After IPTG induction, the expression of candidate genes in BL21 was confirmed and analyzed by SDS-PAGE (Figure S2). After 30 min of heat treatment at 50 °C, the colony growth of each dilution time of the *E. coli* BL21 transformed by pET28a was inhibited, and the average survival rate was only 14.46%. Whereas the BL21 cells with pET28a-*AnAFP* grew better, with an average survival rate of 85.29%. Notably, the BL21 cells with pET28a-*AnAFPΔA* was similar to BL21 with pET28a, the average survival was 32.41%. However, the BL21 cells with pET28a-*AnAFPΔK*, pET28a-*AnAFPΔN*, and pET28a-*AnAFPΔS* was similar to that transformed by pET28a-*AnAFP*, and average survival rates were 84.85%, 95.75% and 95.85%, respectively (Figs. 2 and S3), suggesting that A domain as ice crystal binding domain was also related to thermostable function of AnAFP protein.

### Expression of mutant genes increase heat sensitivity of *Arabidopsis*

**Figure 3:**
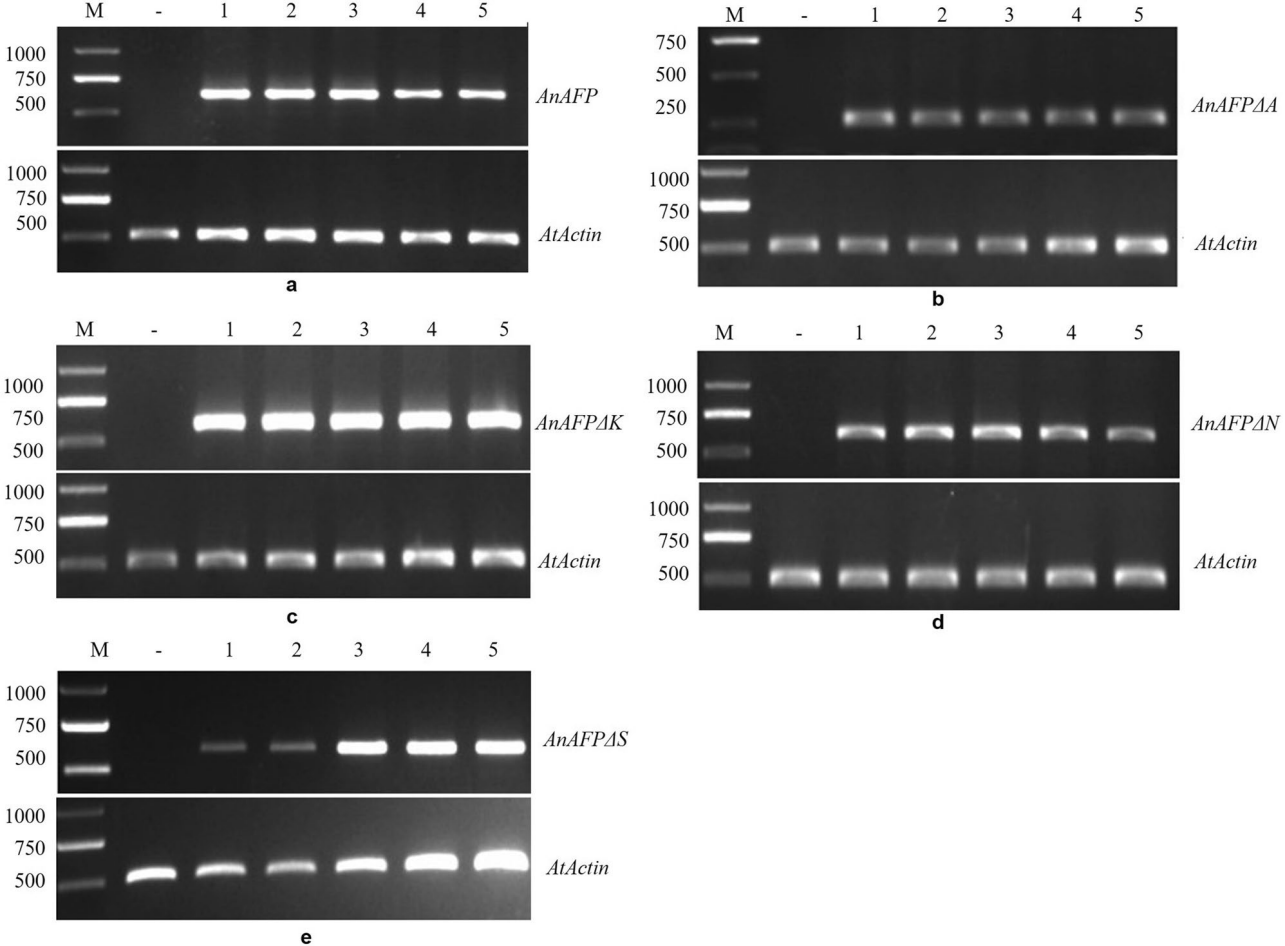
Ectopic expression of *AnAFP* (**a**), *AnAFPΔA* (**b**), *AnAFPΔK* (**c**), *AnAFPΔN* (**d**), *AnAFPΔS* (**e**) genes in T_3_ transgenic *Arabidopsis* by RT-PCR. M: DNA molecular weight marker DL2000; –: Untransformed mutant; 1, 2, 3, 4, 5 indicates independent transgenic line.

After screening with 50 mg/L kanamycin, the five homozygous lines for every gene were identified by PCR amplification of *AnAFPΔA*, *AnAFPΔK*, *AnAFPΔN* and *AnAFPΔS*, respectively (Figure S4), indicating that these genes were integrated into the genome of Arabidopsis. Subsequently, the ORF of *AnAFPΔA*, *AnAFPΔK*, *AnAFPΔN* and *AnAFPΔS* were amplified by RT-PCR from the T_3_ lines of transgenic lines, but not in untransformed mutant. Likewise, the specific fragment of *AtActin* was amplified both in transgenic lines and untransformed mutant (Figs. 3 and S5), indicating the ectopic expression of candidate genes in transgenic plants.

**Figure 4:**
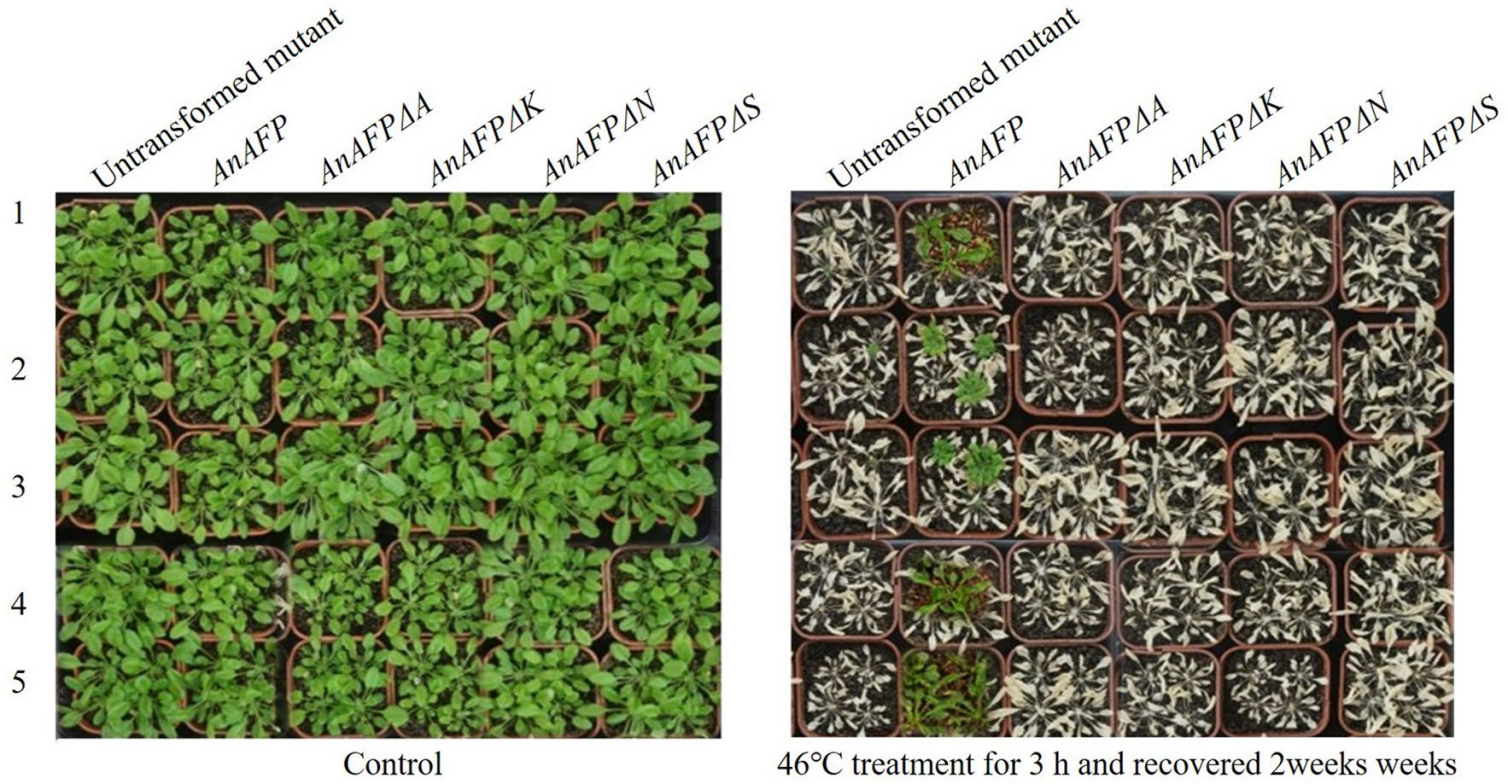
Phenotype of T_3_ transgenic lines and untransformed mutant under heat treatment. 1, 2, 3, 4, 5 indicates independent transgenic line. Five T_3_ lines were planted in pots, and grown in green house at 22 ℃ and 60–70% relative humidity under a 10 h light/14 h dark photoperiod. One-month-old seedlings were used for heat-shock treatment at 46 °C for 3 h, and recovered for 2 weeks at 22 ℃, and investigated for phenotype.

As shown in Fig. 4, before heat treatment, the transgenic lines and untransformed mutants grew vigorously. After 46 °C treatment for 3 h and then recovered 2 weeks, the transgenic lines transformed by *AnAFPΔA*, *AnAFPΔK*, *AnAFPΔN* and *AnAFPΔS* and untransformed mutants all died. However, a few plants of transgenic line transformed by *AnAFP* still survived. This result suggests that ectopic expression of *AnAFP* improves heat tolerance of heat sensitive Arabidopsis mutant. However, deletion of any one of A, K, N and S domains will lead to loss of heat resistance function of AnAFP protein.

### Deletion of domains K and N alters subcellular localization

**Figure 5:**
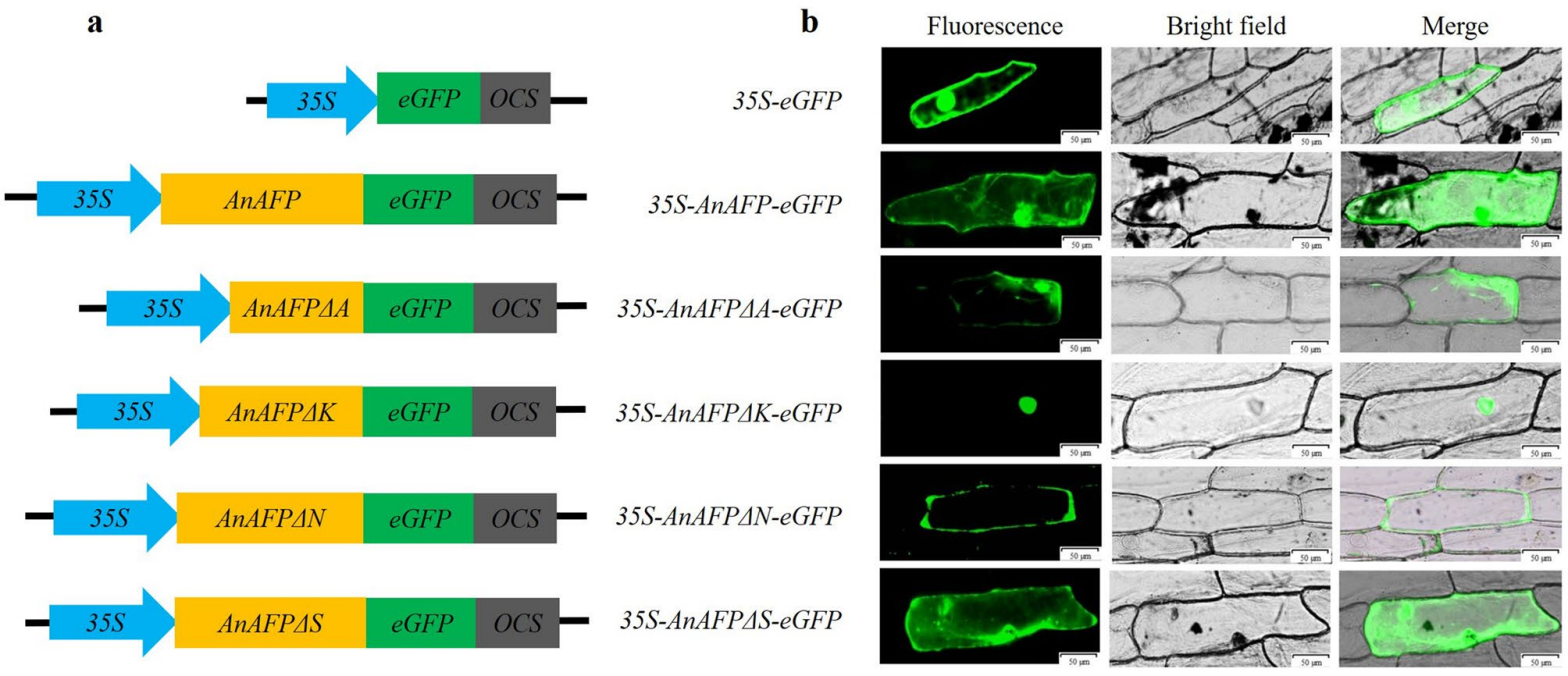
Subcellular localization of AnAFP and its deletion mutants in onion epidermal cells. (**a**) The diagram of vector for transient expression. (**b**) Green fluorescence observed by confocal microscopy. Scale bars = 50 µm.

Subcellular localization results showed that green fluorescence signals were observed both in cytoplasm and nucleus of onion epidermal cells transformed by *35S*-*eGFP*, *35S*-*AnAFP*-*eGFP*, *35S*-*AnAFPΔA*-*eGFP*, and *35S*-*AnAFPΔS*-*eGFP*, respectively. However, green fluorescence was observed only in the nucleus of onion epidermal cells with *35S*-*AnAFPΔK*-*eGFP*, and only in the cytoplasm of onion epidermal cells with *35S*-*AnAFPΔN*-*eGFP* (Fig. 5). These results suggest that the deletion of domains K and N changes the subcellular localization of AnAFP protein.

### Domain K blocks interaction of AnAFP with AnICE1

**Figure 6:**
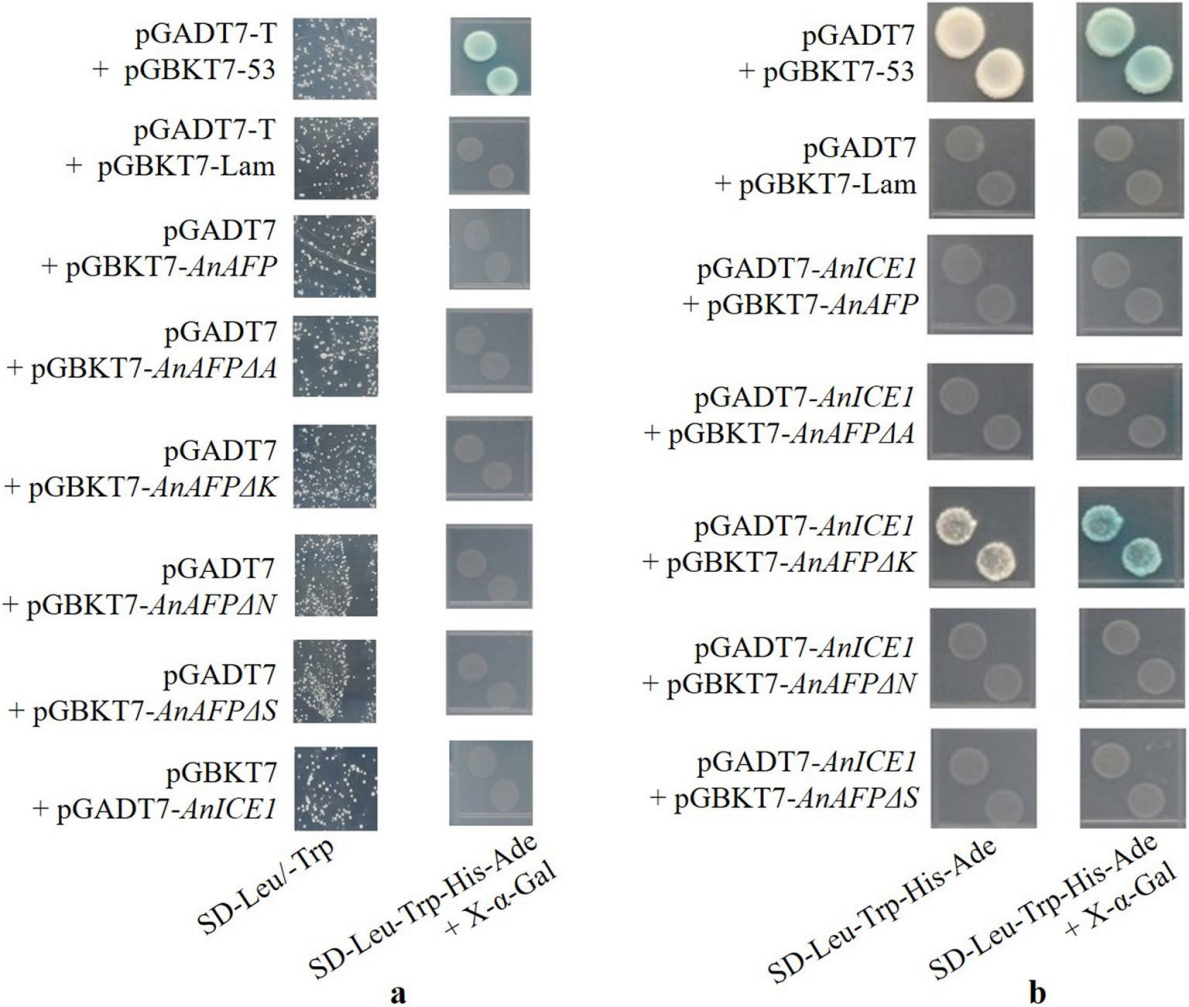
Protein interaction by Y2H. (**a**) The toxicity and autoactivation assay. (**b**) Y2H between AnICE1 and AnAFP, as well as its deletion mutants.

As shown in Fig. 6, the yeast Y2H Gold co-transformed by bait vector and trap vector could grow normally on SD/-Leu-Trp plates, but not grow and not turn blue on SD/-Leu-Trp-His-Ade with X-α-Gal plates except positive control (pGADT7-T + pGBKT7-53), showing that these proteins have no toxicity and no autoactivation in yeast cell. Likewise, the yeast cells with pGBKT7-*AnAFPΔK* and pGADT7-*AnICE1* could grow and be stained to blue on the auxotroph SD/-Leu-Trp-His-Ade with X-α-gal plates, while the cells co-transformed by pGBKT7-*AnAFP*, pGBKT7-*AnAFPΔA*, pGBKT7-*AnAFPΔN*, pGBKT7-*AnAFPΔS*, and pGADT7-*AnICE1* did not grow, suggesting that AnAFPΔK interacts with AnICE1. This result indicates that domain K of AnAFP protein blocks its interaction with AnICE1 protein.

### Endogenous expression of *AnAFP*, *AnICE1* and *AnCBF* genes in response to high temperature stress

**Figure 7:**
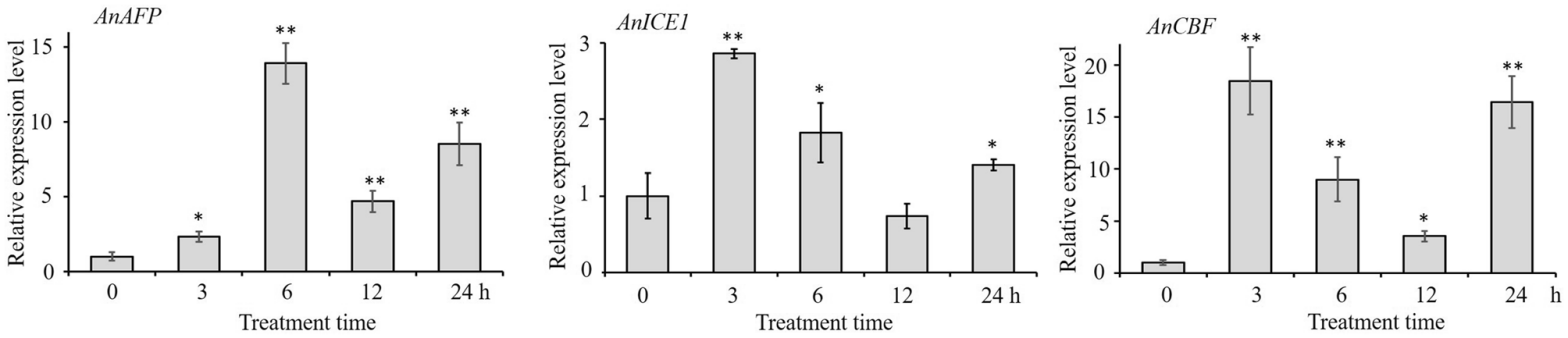
Relative expression levels of *AnAFP*, *AnICE1* and *AnCBF* genes under high temperature (45 °C) treatment. The *AnGAPDH* gene was used as internal reference. The 2^−ΔΔCT^ method of the CFX Manger™ software version 2.0 (Bio-Rad, USA) was used to normalize the expression differentiation between reference gene and investigated genes. The experiment was performed with three replicates. The data were presented as the mean values ± SD.

The results of real-time quantitative PCR (RT-qPCR) showed that the expression of *AnAFP*, *AnICE1* and *AnCBF* genes were significantly upregulated by high temperature stress. The expression of *AnAFP* reached to 13.91 times of control at 6 h of treatment. Meanwhile, at 3 h of treatment, the expression of *AnICE1* and *AnCBF* increased to 2.86 and 18.50 times of control (0 h), respectively (Fig. 7). This result suggests that the upregulated expression of *AnAFP* gene in response to high temperature stress maybe regulated by the signaling pathway of ICE1-CBF-COR.

## Discussion

All AFPs are conserved for their ice crystal binding sites, although difference in their structural characteristics due to their different evolution^24,25^. Under low temperature stress (17 °C for 9 days), the colony growth and average survival rates of the cold-sensitive *E. coli* BX04 with *AnAFPΔA* were significantly poorer and lower than the strains transformed with *AnAFP*, as well as *AnAFPΔK*, *AnAFPΔN* and *AnAFPΔS* mutants (Fig. 1). Under high temperature stress (50 °C for 30 min), the colony growth and average survival rate of *E. coli* BL21 (DE3) transformed by *AnAFPΔA* were also significantly poorer and lower than the strains transformed by AnAFP, as well as *AnAFPΔK*, *AnAFPΔN* and *AnAFPΔS* (Fig. 2). These results confirmed the conserved thermostable function of ice crystal binding domain (domain A) in AnAFP proteins. However, the function of each domain of AnAFP is not indispensable. All T_3_ plants of *Arabidopsis* mutant of KnS type dehydrin *athird11* transformed by *AnAFPΔA*, *AnAFPΔK*, *AnAFPΔN* and *AnAFPΔS* mutants died after high temperature stress (46 °C for for 3 h), while a few T_3_ plants of the same mutant transformed by the *AnAFP* gene survived (Fig. 4). Subcellular localization showed that the mutants without domains A and S were localized in the cytoplasm and nucleus, which was same as AnAFP protein. However, the mutant without domain K was only localized in the nucleus, while the mutant without domain N was localized only in the cytoplasm (Fig. 5). This result suggests that domains K and N determine the subcellular localization of AnAFP protein and affect its function. Similar phenomenon was observed for KnS-type dehydrin ZmDHN13 in maize^26^.

The Y2H result indicates that domain K of AnAFP protein blocks its interaction with AnICE1 proteins (Fig. 6). This result can be explained by the molecular shield model of dehydrin to protect functional proteins^27,28^. The internal disordered structure of dehydrin protein occupies the space between the target proteins and reduces their collision. This kind of shield forms a loose structure around the target protein but not interact with it like the classical molecular chaperone.

**Figure 8:**
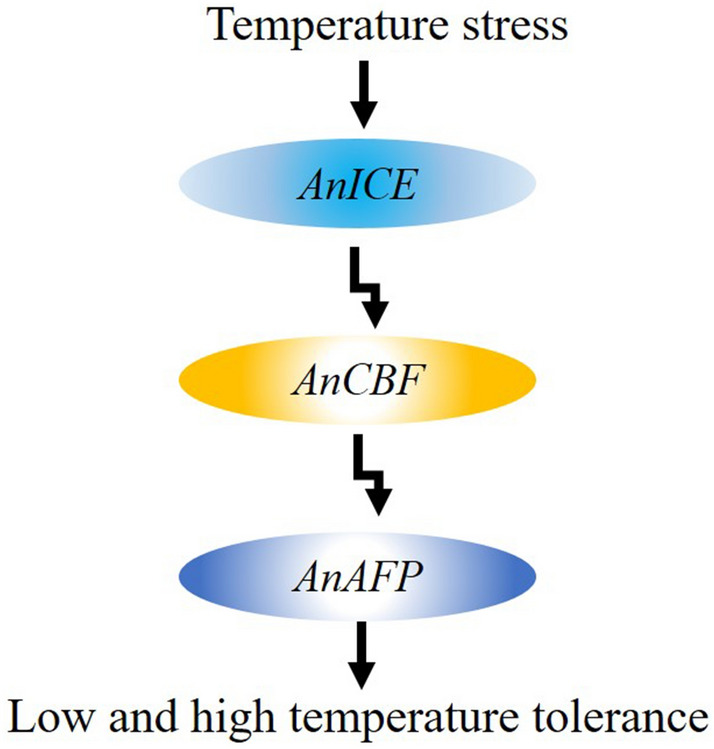
Signaling model of *AnAFP* in *A. nanus* in response to temperature stress.

In our previous study, the AnAFP was found to be a KnS type dehydrin and the *AnAFP* gene was identified as a member of *COR* genes^22^. Dehydrin and *COR* genes can be activated by CBF participating ICE1-CBF-COR pathway^29–32^ In the present study, the RT-qPCR result showed that the endogenous expression of *AnAFP*, *AnICE1* and *AnCBF* genes was significantly upregulated by high temperature stress (Fig. 7). It suggests that high temperature stress also induces the expression of *COR* genes. In response to low and high temperature stress, the endogenous expression of *AnAFP* is probably induced by signaling pathway of ICE1-CBF-COR. In Arabidopsis, the expression of heat shock proteins was also found to be extensively overlapped with non-heat stress response pathways^33^. Therefore, the regulation of the endogenous expression of *AnAFP* in *A. nanus* can be concluded as signaling pathway sketched in Fig. 8.

## Conclusion

In the presents study, we generated *AnAFPΔA*, *AnAFP*ΔK, *AnAFPΔN* and *AnAFP*ΔS, and transformed them into ordinary and cold sensitive strains of *E. coli*, and *Arabidopsis* KS type dehydrin mutant to evaluate their function. It’s confirmed that domain A is a thermal stable functional domain, K and N domains control localization of AnAFP. Likewise, K domain blocks interaction between AnAFP and AnICE1. The expression of *AnAFP*, *AnICE1* and *AnCBF* genes was significantly induced by high-temperature, indicating that the *AnAFP* is likely regulated by ICE1-CBF-COR signal pathway.

## Materials and methods

### Evaluation of *AnAFP* domains in cold resistance of *E. coli*

Two pairs of specific primers (Table S1) with restriction sites of EcoRI/BamHI were designed by using CE Design V1.04 (http://www.downcc.com/soft/281907.html), and used to amplify open reading frame (ORF) of *AnAFP* and four mutants deleting domain A (*AnAFPΔA*), K (*AnAFPΔK*), S (*AnAFPΔS*) and N (*AnAFPΔN*) created in our previous study, respectively. The products were cloned into the EcoRI/BamHI site of prokaryotic expression vector pINIII to generate pINIII-*AnAFP*, pINIII-*AnAFPΔA*, pINIII-*AnAFPΔK*, pINIII-*AnAFPΔS* and pINIII-*AnAFPΔN*, and confirmed by sequencing, respectively.

The re-constructed plasmids were transformed into competent cells of *E. coli* cold-sensitive strain BX04. After confirming by screening with 50 mg/L ampicillin, PCR amplification and sequencing, the positive colonies were transferred into LB liquid medium and incubated at 37 °C until OD6_00_ = 0.5–0.6. According to the methods of Yang et al.^34^ and Deng et al.^21^ with minor modification, the cultured cells were treated at 17 °C for 9 days with three replicates. All samples were diluted by 10^0^ to 10^7^ times, respectively. The 5 μL and 100 μL of them were plated onto LB plates with 50 mg/L ampicillin, incubated at 37 °C for 12 h and photographed, respectively. Before treatment, 5 μL of them were plated onto LB plates with 50 mg/L ampicillin and incubated at 37 °C for 12 h for control. The colonies number with spraying 100 μL cells were counted and used to calculate average survival rates under cold stress.

### Evaluation of *AnAFP* domains in heat resistance of *E. coli*

Two pairs of specific primers (Table S2) with restriction sites of NdeI/HindIII were designed, and used to amplify ORF of *AnAFP*, *AnAFPΔA*, *AnAFPΔK*, *AnAFPΔS* and *AnAFPΔN* using the above plasmids as template, respectively. The products were cloned into *Nde* I/*Hind* III site of prokaryotic expression vector pET28a to generate pET28a-*AnAFP*, pET28a-*AnAFPΔA*, pET28a-*AnAFPΔK*, pET28a-*AnAFPΔS* and pET28a-*AnAFPΔN*, and confirmed by sequencing, respectively.

The recombined plasmids were transferred into competent cells of *E. coli* BL21 (DE3). After confirming by screening with 50 mg/L kanamycin, and PCR amplification and sequencing, the positive colonies were transferred into LB liquid medium and incubated at 37 °C until OD_600_ = 0.5–0.6. The ectopic expression of *AnAFP*, *AnAFPΔA*, *AnAFPΔK*, *AnAFPΔS* and *AnAFPΔN* was induced by 0.5 mmol/L IPTG, detected by sodium dodecyl sulfate–polyacrylamide gel electrophoresis (SDS-PAGE). As described by Li et al.^35^ with minor modification, the induced cells were splinted into six centrifuge tubes, three of them were treated at 50 °C for 30 min, while others were incubated at 37 °C for 30 min, respectively. All samples were diluted by 100 to 104 times, respectively. The 5 μL and 100 μL of them were plated onto LB plates with 50 mg/L kanamycin, incubated at 37 °C for 12 h, and photographed. The colonies number with spraying 100 μL cells were counted and used to calculate average survival rate under heat stress.

### Plasmids reconstruction and *Arabidopsis* transformation

Two pairs of specific primers (Table S3) with restriction sites of BspI/PstI were designed, and used to amplify ORF of *AnAFP*, *AnAFPΔA*, *AnAFPΔK*, *AnAFPΔS* and *AnAFPΔN*, respectively. The products were cloned into *Bsp* I/*Pst* I site of plants expression vector pCAMBIA2300-*35S*-*eGFP* to generate *35S*-*AnAFP*-*eGFP*, *35S*-*AnAFPΔA*-*eGFP*, *35S*-*AnAFPΔK*-*eGFP*, *35S*-*AnAFPΔS*-*eGFP* and *35S*-*AnAFPΔN*-*eGFP*, and confirmed by sequencing, respectively. These plasmids were transferred into competent cells of *Agrobacterium tumefaciens* GV3101 by freeze–thaw method.

After confirming by screening with 50 mg/L rifampicin and 50 mg/L kanamycin on YEB plates, PCR amplification and sequencing, the positive colonies were transferred into liquid medium YEB and incubated at 28 °C until OD6_00_ = 1.0–1.5. The cells were collected by centrifugation at 4 °C and 4000 r/min for 10 min, resuspended and adjusted to OD_600_ = 1.0 with 5% (wt/vol) fresh sucrose solution, added surfactant Silwet L-77 to a concentration of 0.02% (vol/vol), and used to transform *Arabidopsis* mutant of the KnS type dehydrin gene *AtHIRD11* (AT1G54410) by floral-dip method.

### Heat tolerance of transgenic Arabidopsis

As described by Sun et al.^36^, T_1_ seeds were surface-sterilized with 75% ethanol for 1 min and 10% NaClO for 10 min, and plated onto 1/2 MS plates with 50 mg/L kanamycin (Sigma, USA) for screening of transgenic plants, which were used to produce T_2_ generation. The T_2_ plants with 3:1 segregating-ratio to resistance/susceptibility of kanamycin were self-pollinated to generate T_3_. The homozygous lines without segregation were collected from T_3_, and were identified by PCR amplification using the primers (Table S4) for the specific fragments of *AnAFP*, *AnAFPΔA*, *AnAFPΔK*, *AnAFPΔN* and *AnAFPΔS*. The total RNA of every line was extracted by RNA extractor kit (Sangon, China), reacted with RNase-free DNase I, and used to reverse transcribed into cDNA using PrimeScript RT Reagent Kit (TaKaRa, Dalian). The ectopic expression of the transformed genes was identified by reverse transcription PCR (RT-PCR) using the above primers. The *AtActin* gene was amplified and used as reference. Five T_3_ lines were planted in pots, and grown in green house at 22 ℃ and 60–70% relative humidity under a 10 h light/14 h dark photoperiod. One-month-old seedlings were used for heat-shock treatment at 46 °C for 3 h, and recovered for 2 weeks at 22 ℃, and investigated for phenotype.

### Subcellular localization

The *35S*-*AnAFP*-*eGFP*, *35S*-*AnAFPΔA*-*eGFP*, *35S*-*AnAFPΔK*-*eGFP*, *35S*-*AnAFPΔS*-*eGFP* and *35S*-*AnAFPΔN*-*eGFP* plasmids were precipitated onto 50 mg of 0.6 µm gold particles by 2.5 mol/L CaCl_2_ and 0.1 mol/L spermidine, respectively, and used for transformation of intraepidermal cells of onion bulbs by microprojectile bombardment on DuPont PDS 1000/He (Bio-Rad, USA). The green fluorescence signal was observed using laser confocal microscope LSM 800 (Carl Zeiss, Germany).

### Yeast two-hybrid

Two pairs of specific primers (Table S5) with restriction sites of NdeI/BamHI were designed, and used to amplify ORF of *AnAFP*, *AnAFPΔA*, *AnAFPΔK*, *AnAFPΔS* and *AnAFPΔN*, respectively. The products were cloned into NdeI/BamHI sites of yeast two hybrid (Y2H) bait vector pGBKT7 to generate pGBKT7-*AnAFP*, pGBKT7-*AnAFPΔA*, pGBKT7-*AnAFPΔK*, pGBKT7-*AnAFPΔS* and pGBKT7-*AnAFPΔN*, and confirmed by sequencing, respectively. Another pair of specific primers (Table S6) with restriction sites of NdeI/BamHI were designed and used to amplify ORF of *AnICE1* from the cDNA of *A. nanus* seedlings. The products were cloned into Y2H trap vector pGADT7 to generate pGADT7-*AnICE1* and confirmed by sequencing.

The yeast strain Y2H Gold was transformed with every plasmid using Yeastmaker Yeast Transformation System 2 (Clotech, Japan), and used to test of self-activation and toxicity of AnAFP, AnAFPΔA, AnAFPΔK, AnAFPΔN, AnAFPΔS, and AnICE1 proteins. Subsequently, the yeast strain Y2H Gold was co-transformed by each pair of bait vector pGBKT7-*AnAFP*, pGBKT7-*AnAFPΔA*, pGBKT7-*AnAFPΔK*, pGBKT7-*AnAFPΔN*, pGBKT7-*AnAFPΔS*, and trap vector pGADT7-*AnICE1*, respectively. The transformants were screened on the auxotroph SD/-Trp-Leu plates at 30 °C for 3–5 days. The mono-colonies were transferred onto the auxotroph SD/-Leu-Trp-His-Ade plates, incubated at 30 °C for 3–5 days, and strained with X-α-Gal.

### Real-time quantitative PCR

The seeds of *A. nanus* were surface-sterilized with 75% ethanol for 10 min, and planted in soil and grown in green house at 25 °C 12 h light/ 20 °C 12 h dark and 60–70% relative humidity. At the six-leaf stage, the seedlings with same size were treated at high temperature of 45 °C for 0 (negative control), 3, 6, 12, and 24 h with three biological replicates, as described by Yu et al.^37^. The total RNA was extracted, and reverse transcribed into cDNA as above.

For pairs of specific primers (Table S7) were designed, and used to amplify a 150–250 bp fragment for *AnAFP*, *AnAFPΔA*, *AnAFPΔK*, *AnAFPΔN* and *AnAFPΔS*, as well as the internal reference gene *AnGAPDH*. The RT-qPCR was performed using SYBR Green Super Mix (Bio-Rad, USA) by two step real-time PCR cycles (95 °C 30 s; 95 °C 5 s, 50–65 °C 30 s, 39 cycles) in CFX96 Real-Time System (Bio-Rad, USA). The 2^−ΔΔCT^ method was used to normalize the expression differentiation between reference gene and investigated genes^38^.

### Statistical analysis

All experiments were conducted with three replicates. The data are presented as the mean values ±  standard deviation (SD). Statistical difference was analyzed using Student’s t tests.

## Supplementary Information

Supplementary Information.
