# Supplementary material for: Antifreeze protein from Ammopiptanthus nanus functions in temperature-stress through domain A
Source: Sci Rep. 2021 Apr 19;11:8458. doi: 10.1038/s41598-021-88021-0 (PMC8055964; doi:10.1038/s41598-021-88021-0)
Supplement: Supplementary file 1 — Supplementary Information. [file 41598_2021_88021_MOESM1_ESM.docx]

**Figure S1.** Sequence alignments of *AnAFP*, *AnAFPΔA*, *AnAFPΔK*, *AnAFPΔN* and *AnAFPΔS*. (**a**) Nucleic acid sequences. (**b**) Putative protein sequences.


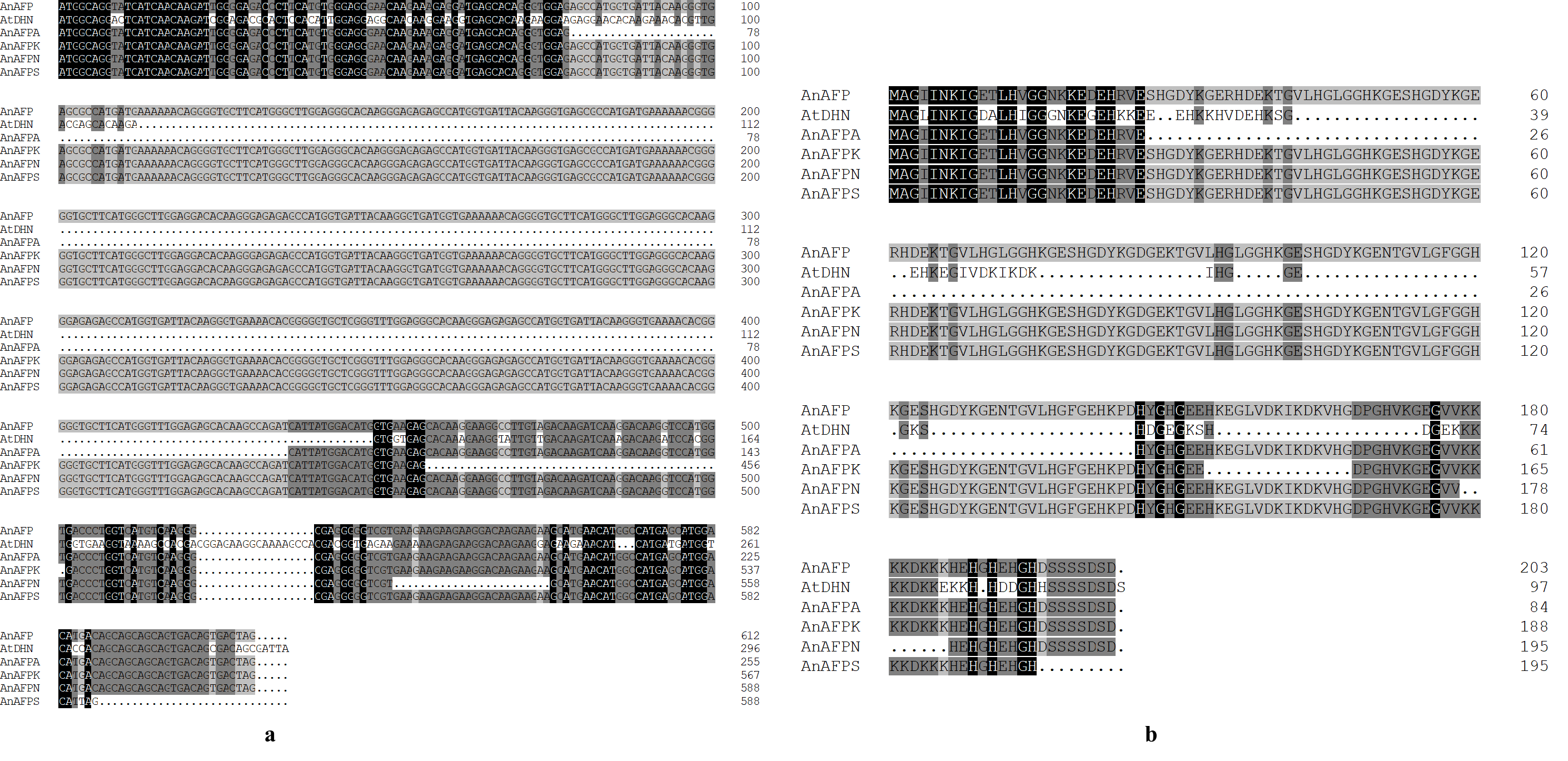


**Figure S2.** The target proteins in BL21 cells analyzed by SDS-PAGE.


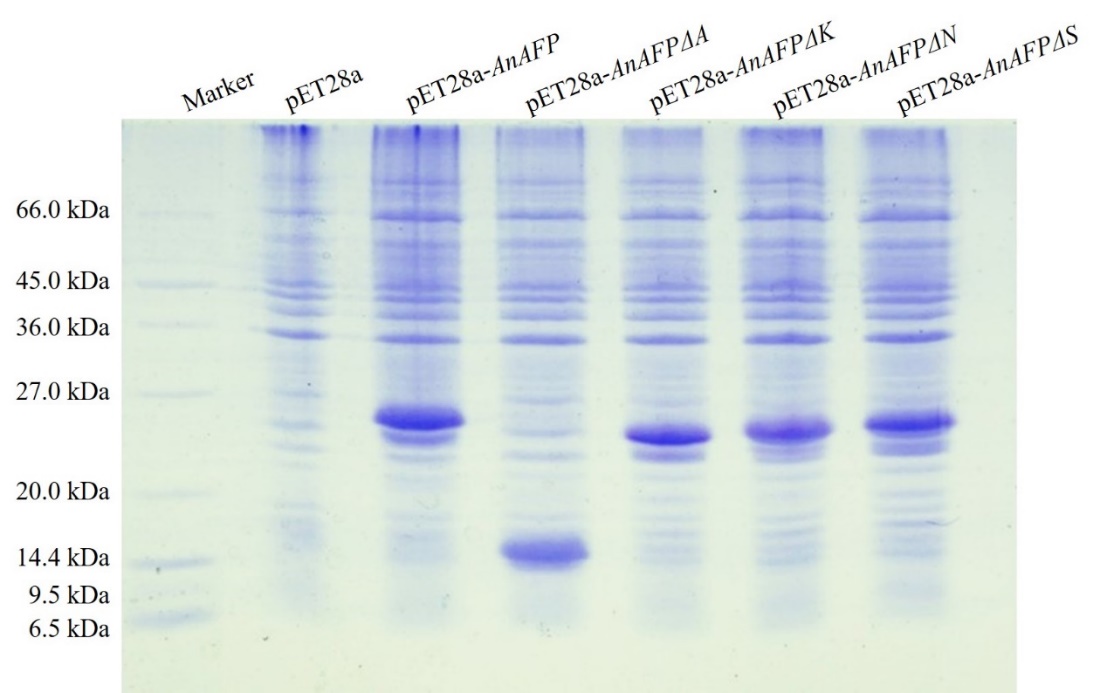


**Figure S3.** The original image of heat phenotype for BL21.


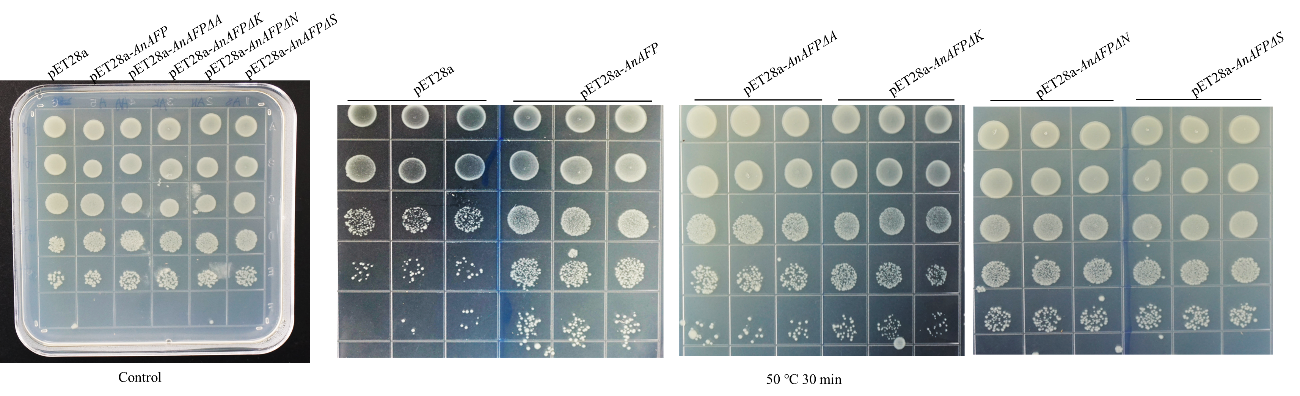


**Figure S4.** PCR identification of transgenic *Arabidopsis* lines. M DNA molecular weight marker DL2000; + Expression vector (positive control); - Untransformed mutant (negative control); 1, 2, 3, 4, 5 independent transgenic lines; (**A**) Transgenic line with *AnAFP*; (**B**) Transgenic line with *AnAFPΔA*. (**C**)Transgenic line with *AnAFPΔK*; (**D**) Transgenic line with *AnAFPΔN*; (**E**) Transgenic line with *AnAFPΔS*.


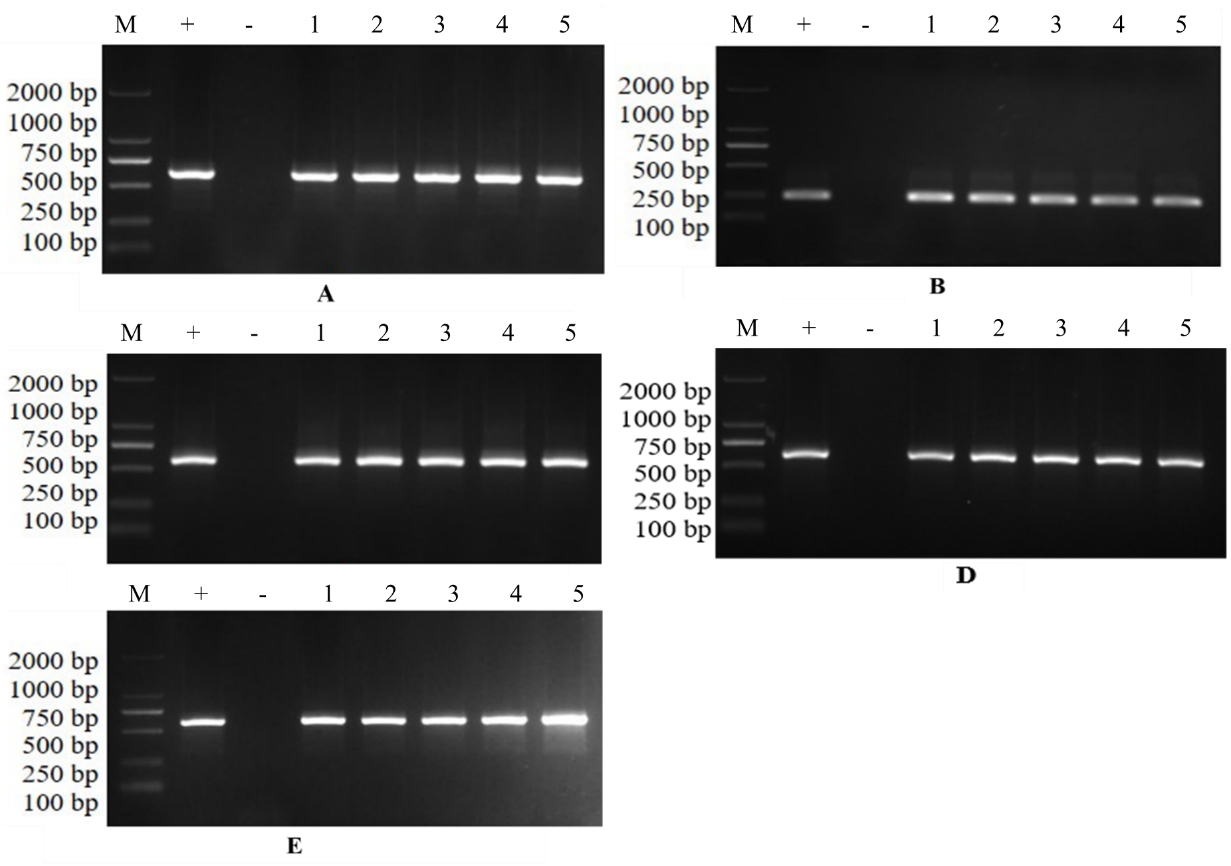


**Figure S5.** The original image of RT-PCR to detect the ectopic expression of *AnAFP*, *AnAFPΔA*, *AnAFPΔK*, *AnAFPΔN*, *AnAFPΔS* genes in T_3_ transgenic *Arabidopsis*.


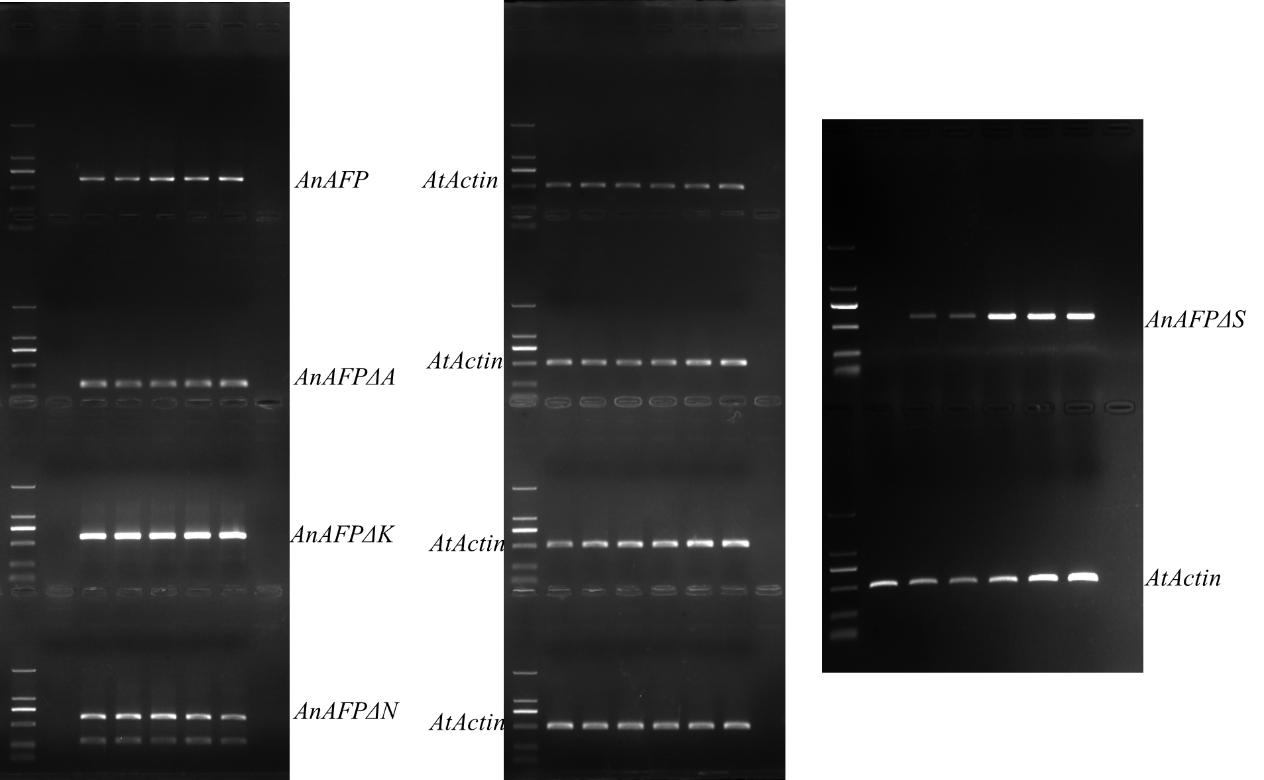


**Figure S6.** The original image of Y2H to detect the interaction of AnAFP, AnAFPΔA, AnAFPΔK, AnAFPΔN, AnAFPΔS and AnICE1.


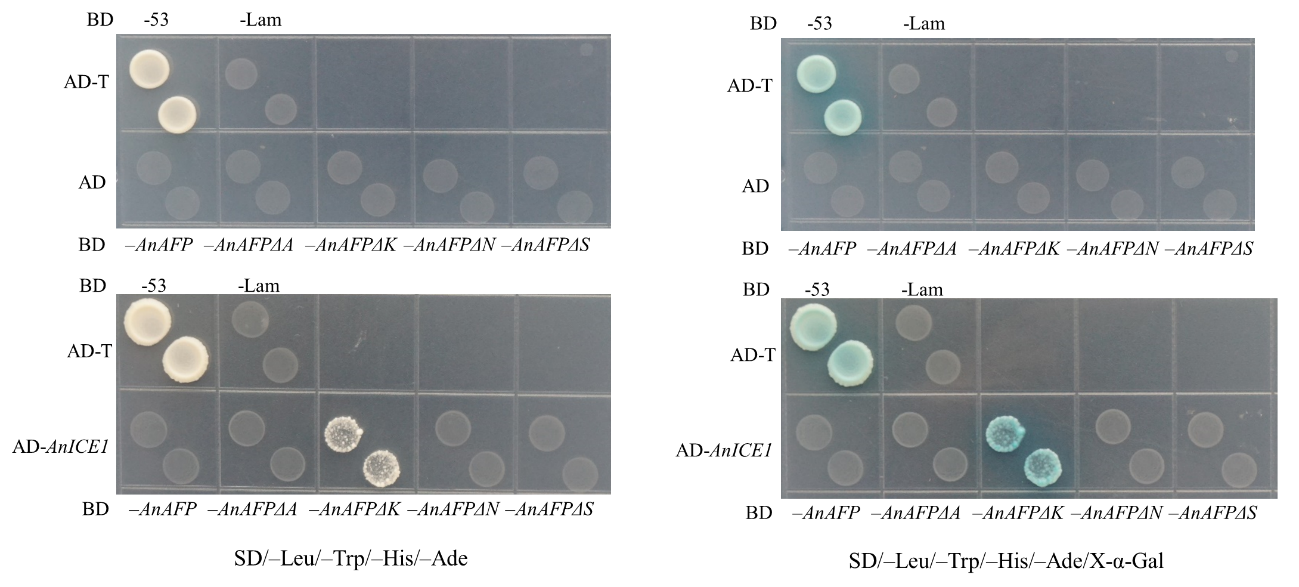


**Table S1.** PCR primers for recombination between vector pINIII and AnAFP gene and its deletion mutants

| Primer | Sequence | Vector | |
| --- | --- | --- | --- |
| pI-AF (*Eco*RI) | 5'-GGAATTCATGGCAGGTATCATCAACAAGATTG-3' | pINIII--*AnAFP*  pINIII-*AnAFPΔA*  pINIII-*AnAFPΔK*  pINIII-*AnAFPΔN* | |
| pI-AR (*Bam*HI) | 5'-CGCGGATCCCTAGTCACTGTCACTGCTGCTGCTG-3' |  |  |
| pI-AF (*Eco*RI) | 5'-GGAATTCATGGCAGGTATCATCAACAAGATTG-3' | pINIII-*AnAFPΔS* | |
| pI-SR (*Bam*HI) | 5'-CGCGGATCCCTAATGTCCATGCTCATGGCCATGT-3' |  |  |
| Note: The underlines represent the restriction enzyme sites *Eco* RI / *Bam* HI. | | |  |

**Table S2.** PCR primers for recombination between vector pET28a and *AnAFP* gene and its deletion mutants

| Primers | Sequence | Vector |
| --- | --- | --- |
| pE-AF | 5'-gtgccgcgcggcagccatatgATGGCAGGTATCATCAACAAGATTG-3' | pET28a-*AnAFP*  pET28a-*AnAFPΔA*  pET28a-*AnAFPΔK*  pET28a-*AnAFPΔN* |
| pE-AR | 5'-ctcgagtgcggccgcaagcttCTAGTCACTGTCACTGCTGCTGC-3' |  |
| pE-AF | 5'-gtgccgcgcggcagccatatgATGGCAGGTATCATCAACAAGATTG-3' | pET28a-*AnAFPΔS* |
| pE-SR | 5'-ctcgagtgcggccgcaagcttCTAATGTCCATGCTCATGGCC-3' |  |
| Note: The lower case letters represent the homologous sequence of the insertion site of the vector. The underlines represent the restriction enzyme sites *Nde* I / *Hind* III. The upper case letters are the sequences homologous to the full-length CDS of the *AnAFP* gene and its deletion mutants. | | |

**Table S3.** PCR primers for recombination between vector pCAMBIA2300 and *AnAFP* gene as well as its deletion mutants

| Primer | Sequence | Vector |
| --- | --- | --- |
| pC-AF (*Bsp*1407I) | 5'-cggcatggacgagctgtacaAGATGGCAGGT  ATCATCAACAAGA-3' | pCAMBIA2300-*AnAFP*  pCAMBIA2300-*AnAFPΔA*  pCAMBIA2300-*AnAFPΔK*  pCAMBIA2300-*AnAFPΔN* |
| pC-AR (*Pst*I) | 5'-cctggcatgcctgcagCTAGTCACTGTCAC  TGCTGCTGCTG-3' |  |
| pC-AF (*Bsp*1407I) | 5'-cggcatggacgagctgtacaAGATGGCAGGT  ATCATCAACAAGA-3' | pCAMBIA2300-*AnAFPΔS* |
| pC-SR (*Pst*I) | 5'-cctggcatgcctgcagCTAATGTCCATGCT  CATGGCCATGTT-3' |  |
| Note: The lower case letters represent the homologous sequence of the insertion site of the vector. The underlines represent the restriction enzyme sites *Eco* RI / *Bam* HI. The upper case letters are the sequences homologous to the full-length CDS of the *AnAFP* gene and its deletion mutants. | | |

**Table S4.** Primers for PCR identification of transgenic *Arabidopsis* lines

| Primer | Sequence | Gene |
| --- | --- | --- |
| AF | 5'-ATGGCAGGTATCATCAACAAGATTG-3' | *AnAFP*, *AnAFPΔA*, *AnAFPΔK*, and *AnAFPΔN* |
| AR | 5'-CTAGTCACTGTCACTGCTGCTGCTG-3' |  |
| AF | 5'-ATGGCAGGTATCATCAACAAGATTG-3' | *AnAFPΔS* |
| SR | 5'-CTAATGTCCATGCTCATGGCCATGT-3' |  |

**Table S5.** PCR primers for recombination between Y2H bait vectors pGBKT7 and *AnAFP* gene as well its deletion mutants

| Primer | Sequence | Vector |
| --- | --- | --- |
| pGB-AF (*Nde* I) | 5'-tcagaggaggacctgcatatgATGGCAGGTATCAT  CAACAAGATTG-3' | pGBKT7-*AnAFP*  pGBKT7-*AnAFPΔA*  pGBKT7-*AnAFPΔK*  pGBKT7-*AnAFPΔN* |
| pGB-AR (*Bam* HI) | 5'-ccgctgcaggtcgacggatccCTAGTCACTGTCAC  TGCTGCTGC-3' |  |
| pGB-AF (*Nde* I) | 5'- tcagaggaggacctgcatatgATGGCAGGTATCAT  CAACAAGATTG-3' | pGBKT7-*AnAFPΔS* |
| pGB-SR (*Bam* HI) | 5'-cgctgcaggtcgacggatccCTAATGTCCATGCTC  ATGGCC-3' |  |
| Note: The lower case letters represent the homologous sequences of the insertion site of the bait vector pGBKT7. The underlines represent the restriction enzyme sites *Nde* I / *Hind* III. The upper case letters are the sequences homologous to the full-length CDS of the *AnAFP* gene and its deletion mutants. | | |

**Table S6.** PCR primers for recombination between Y2H trap vector pGADT7 and *AnICE1* gene

| Primer | Sequence | Vector |
| --- | --- | --- |
| pGA-IF (*Nde* I) | 5'-gtaccagattacgctcatatgATGATGTCTAGAACA  AACAGTGTTTCTT-3' | pGADT7-*AnICE1* |
| pGA-IR (*Bam* HI) | 5'-cagctcgagctcgatggatccCTAGATCATGCCTT  GGAAGCCA-3' |  |
| Note: The lower case letters represent the homologous sequences of the insertion site of trap vector pGADT7. The underlines represent the restriction enzyme sites *Nde* I / *Hind* III. The upper case letters are the sequences homologous to the full-length CDS of the *AnICE1* gene. | | |

**Table S7.** Primers for RT-qPCR

| Primer | Sequence |
| --- | --- |
| AnGAPDH-F | 5'-AATGGCATTCCGTGTTCCTACT-3' |
| AnGAPDH-R | 5'-CAACTTTCCCTCTGACTCCTCC-3' |
| AnAFP-F | 5'-TTGGAGAGCACAAGCCAGAT-3' |
| AnAFP-R | 5'-TAGTCACTGTCACTGCTGCTG-3' |
| AnICE1-F | 5'-CTGTGCTTCAGCTTCATGGC-3' |
| AnICE1-R | 5'-CTACAGGGACATGGTGGAGC-3' |
| AnCBF-F | 5'-AAGACCAGGATTTGGCTCGG-3' |
| AnCBF-R | 5'-TCATCCGTCTCCGACTCCTT-3' |
